# 自身免疫性溶血性贫血的治疗

**DOI:** 10.3760/cma.j.cn121090-20231027-00236

**Published:** 2024-06

**Authors:** 文如 岳, 婷 吴, 小钦 王

**Affiliations:** 复旦大学附属华山医院，上海 200040 Huashan Hospital Affiliated to Fudan University, Shanghai 200040, China

## Abstract

自身免疫性溶血性贫血（AIHA）是自身抗体和（或）补体吸附于红细胞表面，通过抗原抗体反应加速红细胞破坏而引起的一种溶血性贫血，根据自身抗体类型及抗原抗体反应的最佳温度分为温抗体型、冷抗体型及混合型。糖皮质激素±利妥昔单抗是温抗体型AIHA（wAIHA）的一线治疗方法，脾切除术曾是复发或难治wAIHA的首选二线治疗。但由于各种并发症的发生，利妥昔单抗逐渐成为糖皮质激素治疗失败患者的首选。其他可选治疗包括免疫抑制剂、血浆置换等。糖皮质激素和脾切除术不适用于冷抗体型AIHA，利妥昔单抗单药或联合苯达莫司汀常作为其一线治疗。补体抑制剂苏替利单抗（Sutimlimab）目前已被批准用于治疗冷凝集素病（CAD）。近年来，许多新兴的治疗方案如针对B细胞、浆细胞药物，补体系统抑制剂和抑制吞噬作用的药物等，在有效性和安全性方面展现出巨大潜力，为AIHA的临床治疗提供了新的视角。

## Abstract (en)

Autoimmune hemolytic anemia（AIHA）is characterized by the accelerated destruction of erythrocytes due to the presence of antibodies and/or complement that bind to antigens on erythrocytes. It can be subdivided into warm, cold or mixed AIHA based on the type of autoantibody and the optimal temperature of antigen-antibody reaction. Glucocorticoid with or without rituximab is the first-line treatment of warm AIHA（wAIHA）, and splenectomy was once the preferred second-line treatment for relapsed or refractory wAIHA. However, due to the various complications of splenectomy, rituximab has gradually become the preferred treatment for patients who have failed glucocorticoid therapy. Other available treatments including immunosuppressants and plasma exchange can be chosen. Rituximab with or without bendamustine is generally taken as the first-line regimen for cold autoimmune hemolytic anemia（cAIHA）, while glucocorticoid and splenectomy are ineffective. Sutimlimab, a kind of complement inhibitor, has been approved for the treatment of cold agglutinin disease（CAD）. In recent years, many new drugs have emerged as treatment options for AIHA. Emerging therapies, including B-cell-directed therapies, plasma cell-directed therapies, complement inhibitors, and phagocytosis inhibition, provide a new perspective for AIHA therapy, showing great potential for clinical applications

自身免疫性溶血性贫血（Autoimmune hemolytic anemia, AIHA）是一种少见的血液系统疾病，是针对红细胞抗原的自身抗体引起的异常免疫反应，目前国内尚无其流行病学数据，国外资料显示AIHA的年发病率为（0.8～3）/10万[1]。根据自身抗体与红细胞反应的最适温度可分为温抗体型（wAIHA）、冷抗体型（cAIHA）和混合型。其中cAIHA包括冷凝集素病（cold agglutinin disease, CAD）、冷凝集素综合征（cold agglutinin syndrome, CAS）及阵发性冷性血红蛋白尿症（paroxysmal cold hemoglobinuria, PCH）。CAD是克隆性淋巴细胞增殖性疾病，是一种定义明确的临床病理实体，而CAS通常继发于其他的疾病，如特定感染、自身免疫性疾病及恶性肿瘤。PCH是一种极为罕见的AIHA，主要见于幼儿，表现为病毒性疾病后的急性溶血性贫血[2]–[3]。AIHA的发病机制尚不是很明确，目前国内治疗主要依赖于糖皮质激素和利妥昔单抗，越来越多的靶向治疗药物的出现为AIHA的治疗提供了更多选择，目前已有一些新型药物进入临床试验阶段。

一、发病机制

目前AIHA的发病机制尚未完全明确，但外来抗原和自身抗原间的分子模拟、中枢和外周耐受失调、细胞因子轴破坏、潜在疾病的病理机制和遗传因素被认为是AIHA发生的主要原因。其中B细胞和T细胞的作用尤为重要[4]。

wAIHA抗体类型主要为多克隆性IgG，最适反应温度为37 °C[1],[5]。原发性wAIHA通常涉及以下几种溶血机制：巨噬细胞表面的FcR与IgG结合，导致红细胞破坏或部分红细胞膜被吞噬，形成不易变形的球形红细胞，进入脾脏后被困于脾窦，进而被吞噬清除造成溶血；此外，IgG可以激活补体系统产生C3b，C3b调理的红细胞被肝脏的巨噬细胞捕获吞噬；在严重的情况下，补体激活会导致膜攻击体复合物（C5b-9）的形成并产生血管内溶血，不过这种情况比较少见[6]–[7]。

CAD主要发病机制为克隆性B细胞增殖和补体激活，抗体类型主要为单克隆性IgM，其与红细胞表面的Ⅰ抗原结合可使红细胞发生凝集，最佳反应温度为0～4 °C[3],[5]。IgM是一种强补体激活剂，可以与C1q结合激活经典补体途径产生C3b，当血液温度升高到37 °C时，IgM从红细胞表面脱落，而C3b仍固定在红细胞上，C3b调理的红细胞被肝脏吞噬系统破坏，导致血管外溶血，这是CAD的主要溶血机制；由于红细胞膜上CD55和CD59的表达，通常情况下膜攻击体复合物不会被激活，但在少数严重病例中，可能会发生终末补体途径导致的血管内溶血[7]。

二、当前临床治疗方案

（一）wAIHA

1. 糖皮质激素：糖皮质激素为wAIHA的一线治疗方案。据研究，口服泼尼松治疗初始有效率为70％～80％，但只有20％～30％的患者在首次治疗后获得持久缓解，大部分患者会有一个慢性复发的过程，需要泼尼松或其他药物维持治疗[8]–[9]。

2. 利妥昔单抗：利妥昔单抗是一种抗CD20单克隆抗体，常为复发/难治（Relapsed/Refractory, R/R）AIHA患者的二线治疗或与糖皮质激素联合应用作为一线方案。其单药治疗的有效率为70％～80％，大多数患者在首次给药后4周内出现反应[10]。一项由64例患者组成的Ⅲ期前瞻性临床试验证明，与泼尼松单药相比，利妥昔单抗和泼尼松联合治疗的患者有更高的反应率和更长的无复发生存时间[11]。另一项Ⅲ期随机双盲安慰剂对照临床试验显示利妥昔单抗与泼尼松联合使用者的1年（75％和31％）和2年（63％和19％）总有效率（ORR）均高于单用泼尼松者[12]。因此泼尼松联合利妥昔单抗亦可作为一线治疗方案。

3. 脾切除术：在利妥昔单抗广泛应用前，由于没有更多的选择，脾切除术常作为二线治疗方案，其初始反应率为60％～70％[13]。一项脾切除术后的回顾性分析纳入了37例AIHA患者，分析结果显示绝大多数AIHA患者的贫血和溶血症状均获得了短期或长期缓解[14]，但由于脾切除术后严重感染、血栓栓塞等并发症的产生，以及越来越多新型药物的出现，接受脾切除术治疗的AIHA患者越来越少。

4. 免疫抑制剂：对于利妥昔单抗难治且不适合或拒绝脾切除术的患者亦可考虑使用免疫抑制剂如硫唑嘌呤、环磷酰胺、霉酚酸酯、环孢素A等[5],[8]，然而这些药物的疗效有限且有一定的毒性反应，需要谨慎选择。

（二）CAD

糖皮质激素对CAD的治疗作用有限，且在用药剂量较大时才能观察到疗效。由于CAD患者红细胞的清除主要发生在肝脏，所以脾切除术治疗效果不佳。基于其发病机制，治疗主要针对B细胞克隆和补体激活。

1. 热保护：避免患者暴露于寒冷环境，注意保护患者的头部、面部和四肢远端，避免输注冷性液体，以免病情发生恶化[2]。

2. 针对B细胞克隆：挪威和丹麦的两项研究显示利妥昔单抗单药治疗CAD的ORR约为50％，中位应答时间为11个月，但几乎所有患者都是部分应答[15]–[16]。因此，含利妥昔单抗的联合治疗方案应运而生，在2010年的一项前瞻性研究中，29例患者接受了利妥昔单抗联合氟达拉滨的治疗，ORR为76％，但有41％的患者出现了3～4级血液学毒性[17]。10年后的再评估结果发现该治疗方案患者的ORR为62％，完全缓解（CR）率38％，中位反应持续时间为77个月，然而晚期恶性肿瘤发生率高达31％[18]。另一项前瞻性研究纳入了45例CAD患者，利妥昔单抗联合苯达莫司汀治疗4个周期，结果显示ORR为71％，CR率40％。其中2例对利妥昔单抗联合氟达拉滨治疗无效的患者经该方案治疗后1例达CR，1例达部分缓解（PR），且该方案的耐受性良好[19]。在3年后重新评估时患者的ORR为78％，CR率53％，估计中位反应持续时间在88个月时仍未达到，晚期恶性肿瘤发生率仅9％[18]。据此，相比于氟达拉滨，利妥昔单抗联合苯达莫司汀治疗患者有更高的反应率和反应持续时间，且毒性更低，因此利妥昔单抗联合苯达莫司汀常作为严重CAD患者的一线治疗方案。

3. 针对补体系统：苏替利单抗（Sutimlimab）是一种针对经典补体途径中C1s的人源化单克隆抗体，已成为首个获批用于CAD患者的补体抑制剂[20]。一项开放标签的Ⅲ期CARDINAL研究，纳入了24例近期有输血史的CAD患者，研究结果表明苏替利单抗可以迅速改善溶血、增加HGB水平并减轻疲劳[21]。该研究的2年随访结果表明长期使用苏替利单抗治疗可以持续改善HGB及溶血指标、缓解疲劳，减轻CAD患者的疾病负担[22]–[23]。但在停止使用苏替利单抗9周后进一步随访时发现HGB的平均水平较治疗期间有所下降，而溶血性标志物和C4水平也有所上升，对经典补体途径的抑制作用也减弱[23]–[24]，故使用苏替利单抗治疗的CAD患者应长期服药。此外，一项安慰剂对照的CADENZA研究，以近期无输血史的CAD患者为研究对象，也表明苏替利单抗可以改善HGB水平、减轻疲劳[20]。鉴于其较为可观的治疗前景，苏替利单抗目前已被美国等国家批准用于利妥昔单抗治疗无效的CAD患者。

三、试验性药物

随着对AIHA发病机制的深入研究，越来越多的新兴治疗药物随之出现，主要包括针对B细胞、浆细胞、补体系统及抑制吞噬作用等，目前的新兴药物及治疗策略仍处于不同的临床研究阶段（表1）。

**表1:** 自身免疫性溶血性贫血（AIHA）新药及治疗策略临床试验汇总

靶向药物分类	靶点	药物	注册号	试验阶段	入组例数	疾病类型	当前状态
针对B细胞	CD20	利妥昔单抗+泼尼松	NCT01345708	Ⅱ期	23	AIHA	ORR 82.6%
针对B细胞	CD20	利妥昔单抗+氟达拉滨	NCT00373594	Ⅱ期	30	CAD	CR 21%；PR 55%
针对B细胞	CD20	利妥昔单抗+苯达莫司汀	NCT02689986	Ⅱ期	43	CAD	ORR 71%
针对B细胞	CD20	利妥昔单抗+阿仑珠单抗	NCT00749112	Ⅱ/Ⅲ期	19	难治、复发或依赖类固醇激素治疗的AIHA和特发性血小板减少性紫癜	CR 58%
针对B细胞	PI3K δ	Parsaclisib	NCT03538041	Ⅱ期	25	AIHA	CR 32%；PR 64%
针对B细胞	PI3K δ	Parsaclisib	NCT05073458	Ⅲ期	13	wAIHA	正在进行中
针对B细胞	PI3K δ	Linperlisib	NCT05676697	Ⅰ期	20	R/R AIHA	招募中
针对B细胞	BTK	Zanubrutinib	NCT05922839	Ⅱ期	22	R/R wAIHA	尚未招募
针对B细胞	BTK	伊布替尼	NCT04398459	Ⅱ期	18	R/R AIHA	招募中
针对B细胞	BTK	伊布替尼	NCT05694312	Ⅱ期	45	伴有CLL/SLL的AIHA或CLL样MBL	正在进行中
针对B细胞	BTK	Rilzabrutinib	NCT05002777	Ⅱ期	20	wAIHA	招募中
针对B细胞	BTK	阿卡替尼	NCT04657094	Ⅱ期	4	伴有CLL的R/R AIHA	正在进行中
针对B细胞	mTOR	西罗莫司	NCT05925023	Ⅱ期	22	R/R wAIHA	招募中
针对B细胞	CD19+FcγRIIb	Obexelimab	NCT05786573	Ⅲ期	134	wAIHA	尚未招募
针对B细胞	BAFF-R	Ianalumab	NCT05648968	Ⅲ期	90	wAIHA	招募中
针对B细胞	CD19/BCMA	CD19/BCMA CAR-T	NCT05263817	早Ⅰ期	75	AIHA	招募中
针对浆细胞	蛋白酶体	硼替佐米	NCT01696474	Ⅱ期	21	CAD	CR 15.8%；PR 15.8%
针对浆细胞	蛋白酶体	利妥昔单抗+硼替佐米	NCT04083014	Ⅱ期	43	RR AIHA	招募中
针对浆细胞	CD38	达雷妥尤单抗	NCT05004259	Ⅰ期	10	RR AIHA	招募中
补体抑制剂	补体C1s	Riliprubart	NCT04802057	Ⅰ期	9	CAD	正在进行中
补体抑制剂	补体C1s	Sutimlimab	NCT05791708	观察性研究	400	CAD	招募中
补体抑制剂	补体C1s	Sutimlimab	NCT03347396	Ⅲ期	24	CAD	ORR 54.2%
补体抑制剂	补体C1s	Sutimlimab	NCT03347422	Ⅲ期	42	CAD	ORR 72.7%
补体抑制剂	补体C3	Pegcetacoplan	NCT05096403	Ⅲ期	57	CAD	招募中
补体抑制剂	补体C5	依库珠单抗	NCT01303952	Ⅱ期	13	CAD	LDH从572 U/L降至334 U/L
抑制吞噬作用	SYK	福他替尼	NCT02612558	Ⅱ期	26	wAIHA	ORR 46%
抑制吞噬作用	SYK	福他替尼	NCT04138927	Ⅲ期	90	wAIHA	正在进行中
抑制吞噬作用	SYK	福他替尼	NCT03764618	Ⅲ期	90	wAIHA	ORR 46.7%
抑制吞噬作用	SYK	Sovleplenib	NCT05535933	Ⅱ/Ⅲ期	110	wAIHA	正在进行中
抑制吞噬作用	FcRn	Nipocalimab	NCT04119050	Ⅱ/Ⅲ期	111	wAIHA	招募中
抑制吞噬作用	FcRn	巴利托单抗	NCT04253236	Ⅱ期	5	wAIHA	ORR 20%

**注** RR AIHA：复发难治性AIHA；ORR：总反应率；CR：完全缓解；PR：部分缓解；CAD：冷凝集素病；wAIHA：温抗体型自身免疫性溶血性贫血；BTK：布鲁顿酪氨酸蛋白激酶；CLL/SLL：慢性淋巴细胞白血病/小淋巴细胞淋巴瘤；CLL样MBL：慢性淋巴细胞白血病样单克隆B淋巴细胞增多症；mTOR：哺乳动物雷帕霉素靶蛋白；BAFF-R：B细胞活化因子受体；BCMA：B细胞成熟抗原；CAR-T：嵌合抗原受体T细胞；SYK：脾脏酪氨酸激酶；FcRn：新生儿可结晶片段受体

（一）针对B细胞

1. 磷脂酰肌醇-3-激酶（PI3K）抑制剂：Parsaclisib是一种PI3Kδ抑制剂，能够减少B细胞产生自身抗体。一项前瞻性Ⅱ期研究，纳入25例R/R AIHA患者，在使用Parsaclisib治疗后，患者的ORR为64％，CR率为32％，其中wAIHA患者反应率更高：ORR达75％、CR率为50％。但其毒性反应也值得关注，在为期12周的初始治疗阶段84％的患者发生不良反应，其中7例与Parsaclisib治疗相关；在延长期，17例患者中15例发生不良反应，其中6例与Parsaclisib治疗相关[25]。因此Parsaclisib的安全性仍需进一步探究。目前该药物正在进行一项Ⅲ期随机双盲临床试验（NCT05073458），以评估Parsaclisib在wAIHA中的有效性和安全性。

2. 布鲁顿酪氨酸蛋白激酶（BTK）抑制剂：一项临床前研究证明，伊布替尼和阿卡替尼能减少AIHA小鼠模型中自身抗体的产生[26]。一项纳入了15例接受伊布替尼治疗的cAIHA（包括4例CAD、11例CAS）患者的回顾性研究，发现几乎所有患者HGB和溶血指标均有所改善，但由于其样本量较少且缺少对照，仍需前瞻性研究进一步证实[27]。此外，一项阿卡替尼治疗慢性淋巴细胞白血病（CLL）合并R/R AIHA有效性的Ⅱ期试验正在进行（NCT04657094），利扎鲁替尼（Rilzabrutinib）对类固醇激素治疗失败的wAIHA患者的疗效和安全性评价的Ⅱ期临床试验也正在招募中（NCT05002777）。

3. 哺乳动物雷帕霉素靶蛋白（mTOR）抑制剂：西罗莫司（Sirolimus）是一种mTOR抑制剂，可以诱导自体活性淋巴细胞凋亡，增加外周血调节性T细胞（Treg）水平，从而抑制自身免疫和炎症性疾病，目前已成功使多例实体器官移植后AIHA患者的溶血症状得到持续缓解[28]。一项评估西罗莫司对R/R wAIHA患者的有效性和安全性的Ⅱ期前瞻性研究正在招募中（NCT05925023）。

（二）针对浆细胞

1. 蛋白酶体抑制剂：研究发现部分患者对利妥昔单抗治疗无效，可能是由于AIHA患者脾脏中存在可以生成抗红细胞抗体的长寿命浆细胞。而硼替佐米可以靶向长寿命浆细胞，进而减少自身抗体的产生[29]。在前瞻性Ⅱ期临床试验GIMEMA研究中，19例CAD患者接受了硼替佐米治疗，CR率和PR率均为15.8％，研究结果表明硼替佐米可使部分CAD患者获益，而且毒性可接受[30]。硼替佐米也可与其他药物联合治疗AIHA，一项小型前瞻性研究，使用低剂量利妥昔单抗联合硼替佐米和地塞米松（LowR-BD）治疗7例wAIHA患者，研究发现LowR-BD方案治疗4周后ORR达85.71％，且患者对治疗方案的耐受性较好[31]。此外，一项评估单剂量利妥昔单抗联合短疗程硼替佐米治疗RR AIHA疗效的临床试验目前正在招募患者（NCT04083014）。尽管如此，由于研究规模较小，可能存在选择偏倚，硼替佐米用于治疗AIHA的证据仍然不足。

2. CD38单克隆抗体：达雷妥尤单抗可以靶向长寿命浆细胞表面的CD38从而减少自身抗体。但目前其效果仅限于一些病例报告和回顾性研究[32]，仍需前瞻性研究进一步证实。一项评估达雷妥尤单抗在R/R AIHA患者中安全性和有效性的Ⅰ期试验正在招募中（NCT05004259）。

（三）补体抑制剂

依库珠单抗（Eculizumab）是一种补体C5抑制剂，可防止膜攻击复合物形成和由此引起的血管内溶血，但不会影响C3b引起的血管外溶血[33]。在一项开放标签的前瞻性Ⅱ期研究中，依库珠单抗能显著降低CAD患者的LDH水平及输血需求，但HGB仅有少量增加[34]。另外在1例严重的特发性wAIHA病例报告中显示在标签外使用依库珠单抗可以快速改善溶血状况，为免疫抑制剂发挥作用提供时间[35]。因此可将其作为类固醇激素、利妥昔单抗等治疗无效时的桥接治疗。

Pegcetacoplan是一种C3抑制剂，在一项Ⅱ期临床试验中对CAD患者表现出良好的改善溶血和贫血的作用，并有较低的毒性[36]，目前Ⅲ期临床试验正在招募中（NCT05096403）。

（四）抑制吞噬作用

1. 脾脏酪氨酸激酶（SYK）抑制剂：福他替尼（Fostamatinib）可以在体内快速转化为R406。R406是IgE和IgG介导的FcR信号激活的有效抑制剂，可以阻断FcR介导的巨噬细胞激活及BCR介导的B淋巴细胞激活[37]，从而抑制吞噬作用和减少抗体产生。一项Ⅱ期、多中心研究评估了至少一种治疗方案失败的成人wAIHA患者对福他替尼的反应性和安全性，24例患者中11例达到了主要终点。应答者中HGB水平及LDH等溶血指标均获得了明显改善[38]。安慰剂对照的Ⅲ期临床试验，纳入了90例既往至少1种wAIHA治疗方案失败的患者，研究表明福他替尼可使35.6％的患者达到主要终点，46.7％的患者HGB有所改善[39]（NCT03764618）。一项开放标签的扩展研究正在进行中（NCT04138927）。

2. 新生儿可结晶片段受体（FcRn）抑制剂：FcRn是IgG的细胞内保护受体，负责从溶酶体降解中拯救IgG，FcRn抑制剂奥诺利单抗（Orilanolimab，SYNT001）通过阻止FcRn与IgG的结合，使IgG降解，降低血液中IgG水平，从而治疗AIHA[40]。尼卡利单抗（Nipocalimab，M281）是一种单克隆IgG1抗FcRn抗体，一项评估尼卡利单抗在wAIHA中的疗效和安全性的Ⅱ/Ⅲ期随机双盲、安慰剂对照试验目前正在招募患者（NCT04119050）。

四、展望

针对B细胞、浆细胞、补体系统及抑制吞噬作用的新兴药物的不断发展为R/R AIHA的诊疗提供了新思路，但仍需进一步研究来证实其疗效和安全性。苏替利单抗已被证明对改善CAD患者贫血和溶血症状、减轻疲劳有较好的疗效，目前已被美国等国家获批用于利妥昔单抗治疗无效的CAD患者。众多联合用药方案的产生也为AIHA患者的治疗带来了新进展，期待未来通过对发病机制和新兴靶点的深入研究为更多患者带来新希望。
